# Vitamina C na síndrome da dor complexa regional em pacientes com fratura distal do rádio

**DOI:** 10.1055/s-0045-1810403

**Published:** 2025-08-25

**Authors:** Fernando Antonio Silva de Azevedo Filho, Roberta Muniz Blunck, Abdouni Y Ali, Patrícia Maria de Moraes Barros Fucs, Ricardo Britto Cotias

**Affiliations:** 1 Hospital do Subúrbio, Salvador, BA, Brasil; 2 Faculdade de Ciências Médicas, Santa Casa de São Paulo, São Paulo, SP, Brasil; 3 Irmandade da Santa Casa de Misericórdia de São Paulo, São Paulo, SP, Brasil

**Keywords:** ácido ascórbico, dor crônica, fraturas do rádio, síndrome da dor regional complexa, ascorbic acid, chronic pain, complex regional pain syndromes, radius fractures

## Abstract

**Objetivo:**

Avaliar o efeito da vitamina C na prevenção da síndrome da dor complexa regional (SDCR) em pacientes com fratura da extremidade distal do rádio (FEDR).

**Métodos:**

Foram incluídos pacientes com FEDR com indicação de tratamento cirúrgico e idade acima de 18 anos, sendo avaliados faixa etária, gênero, dominância, presença de comorbidades, lado acometido, mecanismo do trauma e técnica cirúrgica. Os participantes foram randomizados em dois grupos: placebo ( *N* = 64), que recebeu celulose microcristalina, e intervenção ( *N* = 58), que recebeu 1 g/dia de vitamina C, em dose única durante 60 dias, iniciando no pós-operatório imediato.

**Resultados:**

A incidência de dor neuropática foi em média 17% maior no grupo placebo do que no grupo vitamina C nas semanas avaliadas ( *p* = 0,008). A escala visual análoga (EVA) foi em média 1,22 pontos maior no grupo placebo independente do momento de avaliação ( *p* = 0,001). A SDCR, em 12 e 24 semanas, foi estatisticamente mais frequente no grupo placebo ( *p* = 0,014 e 0,007, respectivamente).

**Conclusão:**

A vitamina C tem efeito na prevenção da dor neuropática e da SDCR, bem como no controle da dor pós-operatória.

## Abstract (en)

**Objective:**

To evaluate the effect of vitamin C in preventing complex regional pain syndrome (CRPS) in patients with fracture of the distal end of the radius (FDER).

**Methods:**

The present study included FDER patients with an indication for surgical treatment and aged over 18 years. We evaluated age group, gender, dominance, presence of comorbidities, affected side, trauma mechanism, and surgical technique. Participants were randomized into 2 groups: placebo ( *N* = 64), which received microcrystalline cellulose, and intervention ( *N* = 58), treated with 1 g/day of vitamin C in a single dose for 60 days, starting immediately after surgery.

**Results:**

The incidence of neuropathic pain was on average 17% higher in the placebo group than in the vitamin C one during the evaluation period ( *p* = 0.008). The average visual analog scale (VAS) for pain was 1.22 points higher in the placebo group regardless of the evaluation moment ( *p* = 0.001). The CRPS at 12 and 24 weeks was statistically more frequent in the placebo group ( *p* = 0.014 and 0.007, respectively).

**Conclusion:**

Vitamin C has an effect on preventing neuropathic pain and CRPS, as well as on controlling postoperative pain.

## Introdução

As fraturas da extremidade distal do rádio (FEDR) são as mais comuns. Apresentam um claro padrão bimodal em crianças menores de 15 anos e adultos com mais de 50 anos, que têm maior risco de fratura. O tratamento inclui redução fechada e imobilização gessada ou cirurgia. 1

No período pós-operatório imediato, a ativação direta de nociceptores à resposta inflamatória e à eventual lesão de estruturas nervosas provoca, do ponto de vista clínico, dor em repouso no local da cirurgia e em região próxima, assim como dor desencadeada pelo toque ou pelo movimento, indicando sensibilização periférica. 2

A síndrome da dor complexa regional (SDCR) é uma complicação caracterizada por dor crônica e persistente com ausência de dano celular. É descrita como dor autonômica e sensorial, motora, trófica e alterações vasomotoras que culminam na disfunção da extremidade. 3 4 A incidência da SDCR é de cerca de 4% dos pacientes com FEDR. 4 Alguns estudos mostram uma variação maior na incidência, de 1 a 37%, com impacto direto na qualidade de vida, bem-estar e diminuição da capacidade de trabalho. 5 6 Varia na apresentação clínica, sendo classificada em tipos I e II, sem ou com evidência de lesão nervosa, respectivamente. 3 4

Vários estudos clínicos recentes mostraram que a administração de vitamina C nos pacientes com SDCR, neuralgia herpética aguda e pós-herpética, além de dor relacionada com câncer apresenta melhora significativa do quadro álgico, contribuindo para a qualidade de vida. Também se constitui um potente antioxidante que neutraliza os radicais livres e reduz o estresse oxidativo, o que pode contribuir para o tratamento da inflamação e dor crônica. Provou ser segura e eficaz no alívio das dores aguda e crônica, incluindo pacientes ambulatoriais, cirúrgicos e oncológicos, podendo moderar a necessidade de opioides. 7 8

A vitamina C tem um papel complexo e multifacetado na modulação da dor, principalmente através de suas propriedades antioxidantes, modulação da inflamação e suporte à saúde dos tecidos conjuntivos. No entanto, mais pesquisas são necessárias para esclarecer completamente o seu impacto direto na dor e definir diretrizes específicas para sua aplicação clínica.

O objetivo do presente estudo foi avaliar a eficácia da vitamina C para prevenção no surgimento de dor neuropática e da SDCR em pacientes com FEDR, submetidos a tratamento cirúrgico e avaliar o seu impacto no controle da dor pós-operatória.

## Materiais e Métodos

Foi realizado um estudo clínico duplo cego randomizado em pacientes com FEDR, submetidos a tratamento cirúrgico atendidos no serviço de ortopedia e traumatologia de um hospital terciário.

A amostra incluiu pacientes com idade acima de 18 anos, admitidos no período de abril de 2023 a abril de 2024, que apresentavam FEDR com indicação de tratamento cirúrgico. Foram excluídos pacientes pediátricos, politraumatizados, com fraturas bilaterais, com insuficiência renal crônica, histórico de cálculo renal, em uso de vitamina C ou polivitamínico, FEDR ipsilateral, com diagnóstico prévio de dor neuropática, história de deficiência da glicose-6-fosfato desidrogenase e hiperolaxúria, alérgicos à vitamina C e os que não aceitaram participar do protocolo de pesquisa.

Foram avaliados os seguintes aspectos clínicos: faixa etária, gênero, dominância, presença de comorbidades, lado acometido, mecanismo do trauma e técnica cirúrgica utilizada. A avaliação radiográfica consistiu em imagens nas incidências anteroposterior e perfil do punho lesionado, foram classificadas utilizando a classificação Arbeitsgemeinschaft für Osteosynthesefragen (AO).

Os participantes foram randomizados em dois grupos usando uma ferramenta de pesquisa online ( https://www.randomizer.org/ ). O grupo placebo ( *N* = 64) recebeu celulose microcristalina e o de intervenção ( *N* = 58) recebeu 1 g/dia de vitamina C, 9 em uma dose, iniciando no pós-operatório imediato e por 60 dias. A medicação foi prescrita por um membro da equipe sem o conhecimento do paciente e do pesquisador principal, sendo este último o responsável pelo acompanhamento durante as 24 semanas.

Todos os pacientes seguiram o mesmo protocolo de analgesia, sendo dipirona 1 g a cada 6 horas a primeira escolha de analgésico comum e, em caso de alergia, paracetamol 750 mg a cada 6 horas, associado a um opioide fraco. No caso dos opioides, a primeira escolha foi tramadol 100 mg a cada 12 horas e, na existência de alergia, codeína 30 mg foi a segunda. O paciente foi orientado a fazer o uso das medicações de acordo com a intensidade da dor.

No momento da admissão, a funcionalidade dos membros superiores foi determinada pelo *Quick Disabilities of the Arm, Shoulder and Hand* (Quick-DASH), 10 composto de 11 questões que avaliam a função física na última semana. A pontuação varia em cada item de 0 a 5 e o escore final varia de 0 a 100. Além disso, todos os pacientes foram avaliados em relação à depressão e ansiedade, utilizando a escala Hospitalar de Ansiedade e Depressão (HAD). 11

As avaliações clínica, funcional e radiográfica foram realizadas em cinco episódios distribuídos em 2, 4, 6, 12 e 24 semanas. O paciente, o cirurgião assistente e o pesquisador principal foram todos cegos para a randomização, sendo o último responsável pelo acompanhamento no seguimento clínico e avaliação das radiografias para cicatrização de fraturas.

Em cada consulta de acompanhamento, foi solicitado aos pacientes que avaliassem a intensidade da dor naquele momento, utilizando a escala visual analógica (EVA), instrumento unidimensional, representado por uma linha com as extremidades numeradas de 0 a 10, significando “nenhuma dor” e “pior dor imaginável,” respectivamente. Também foi verificada a presença de complicações relacionadas ao tratamento empregado, bem como sinais e sintomas da SDCR, utilizando os critérios de Budapeste. 12

Na 12ª semana, para definir a presença de dor neuropática, foi utilizado o questionário *Douleur neuropathique 4* (DN4), composto de sete itens que se referem a sintomas e outros três relacionados ao exame físico. Cada item pontua um, se a resposta for positiva, e zero se negativa. A somatória de pontos maior ou igual a quatro sugere dor neuropática. 13

Após 24 semanas de acompanhamento, os pacientes foram submetidos a nova avaliação clínica, respondendo aos mesmos instrumentos, assim como à dor quantificada com auxílio da EVA.

Aqueles diagnosticados com SDCR foram submetidos a tratamento medicamentoso, sendo usadas pregabalina ou gabapentina, mantendo seguimento no serviço, e foram encaminhados para acompanhamento com equipe de fisioterapia.

Para armazenamento dos dados coletados, utilizou-se o Excel 2013 (Microsoft Corp.) e para realização das análises foi usado the Statistical Package Social Sciences (SPSS, IBM Corp.) para Windows, versão 22.0. Os testes foram realizados com nível de significância de 5%.

As características qualitativas avaliadas foram descritas segundo grupos com uso de frequências absolutas e relativas, enquanto a associação com uso de testes qui-quadrado ou testes exatos (Fisher ou razão de verossimilhanças) foi verificada. As características quantitativas foram descritas segundo grupos com uso de medidas resumo (média, desvio padrão [DP], mediana e quartis), comparadas entre os grupos com uso do teste t de Student não pareado ou do teste de Mann-Whitney. Os escores da EVA e a presença de dor neuropática aos cinco pontos foram descritos segundo grupos e momentos de avaliação com uso de medidas resumo, frequências absolutas e relativas, sendo comparados entre grupos e momentos respectivamente, com uso de equações de estimação generalizadas (EEG) com distribuição normal, função de ligação identidade, distribuição binomial e função de ligação lógica respectivamente, assumindo matriz de correlações autorregressivas de primeira ordem (AR[1]) entre os momentos de avaliação de um mesmo paciente. Os resultados foram seguidos de comparações múltiplas de Bonferroni para avaliar entre quais grupos e momentos ocorreram as diferenças.

O estudo foi avaliado e aprovado pelo Comitê de Ética em Pesquisa (CAAE: 63013922.1.0000.0040).

## Resultados

O universo pesquisado compunha-se de 137 pacientes, dos quais 122 concluíram o acompanhamento por 24 semanas, sendo 55 mulheres (45,1%) e 67 homens (54,9%), com idade média de 48,1 anos. As características de cada grupo encontram-se detalhadas na Tabela 1 .

**Tabela 1:** Descrição das características pessoais e clínicas segundo grupos e resultado dos testes estatísticos

Variável	Grupo		Total ( *N* = 122)	*p* -value
	Placebo ( *N* = 64)	Vitamina C ( *N* = 58)		
**Idade (anos)**				
média ± DP	47,9 ± 15,4	48,3 ± 15,5	48,1 ± 15,4	0,909**
mediana (p25; p75)	47 (37,3; 63,8)	48,5 (36,8; 60,8)	47,5 (37; 63)	
**Sexo, n (%)**				
Feminino	29 (45,3)	26 (44,8)	55 (45,1)	0,957
Masculino	35 (54,7)	32 (55,2)	67 (54,9)	
**Dominância, n (%)**				
Destro	62 (96,9)	54 (93,1)	116 (95,1)	0,422*
Sinistro	2 (3,1)	4 (6,9)	6 (4,9)	
**Comorbidades, n (%)**				
Nega	46 (71,9)	40 (69)	86 (70,5)	0,682#
HAS	6 (9,4)	7 (12,1)	13 (10,7)	
DM	1 (1,6)	4 (6,9)	5 (4,1)	
HAS + DM	5 (7,8)	3 (5,2)	8 (6,6)	
Transtornos psiquiátricos	2 (3,1)	1 (1,7)	3 (2,5)	
Outras	4 (6,3)	3 (5,2)	7 (5,7)	
**Lado lesionado, n (%)**				
Direito	33 (51,6)	22 (37,9)	55 (45,1)	0,131
Esquerdo	31 (48,4)	36 (62,1)	67 (54,9)	
**Mecanismo de trauma, n (%)**				
Baixa energia	27 (42,2)	29 (50)	56 (45,9)	0,387
Alta energia	37 (57,8)	29 (50)	66 (54,1)	
**AO, n (%)**				
Extra-articular	31 (48,4)	13 (22,4)	44 (36,1)	**0,008**
Articular parcial	6 (9,4)	5 (8,6)	11 (9)	
Articular	27 (42,2)	40 (69)	67 (54,9)	
**Tratamento, n (%)**				
Redução fechada - Fio Kirschiner	45 (70,3)	34 (58,6)	79 (64,8)	0,177
RAFI	19 (29,7)	24 (41,4)	43 (35,2)	
**HAD**				
média ± DP	8,1 ± 6,6	7,7 ± 6,4	7,9 ± 6,5	0,768£
mediana (p25; p75)	7 (3; 12)	7 (3; 10)	7 (3; 11)	

**Abreviações:** AO, classificação AO/OTA; DM, diabetes mellitus; DP, desvio padrão; HAD, escala hospitalar de ansiedade e depressão; HAS, hipertensão arterial sistêmica; RAFI, redução aberta e fixação interna.

**Notas:** Teste do qui-quadrado; * Teste exato de Fisher; # Teste da razão de verossimilhanças; ** Teste t de Student não pareado; £ Teste Mann-Whitney.

O comportamento dos grupos foi estatisticamente semelhante para dor neuropática e EVA ao longo dos momentos de avaliação ( *p*_Interação_ > 0,05), a dor neuropática diferiu estatisticamente entre os grupos independente do momento ( *p*_Grupo_ = 0,013), e a escala de dor diferiu em média entre os grupos independente do momento de avaliação ( *p*_Grupo_ = 0,001) e ao longo dos momentos independente do grupo ( *p*_Momento_ < 0,001), como pode ser visto na Tabela 2 .

**Tabela 2:** Descrição da dor neuropática e da EVA segundo grupos, momentos de avaliação e resultado das comparações

Variável	Grupo		p _Grupo_	p _Momento_	p _Interação_
	Placebo ( *N* = 64)	Vitamina C ( *N* = 58)			
**Dor neuropática**			**0,013**	0,091	0,355
**12 semanas**					
DN4 < 4	45 (70,3)	50 (86,2)			
DN4 ≥ 4	19 (29,7)	8 (13,8)			
**24 semanas**					
DN4 < 4	47 (73,4)	53 (91,4)			
DN4 ≥ 4	17 (26,6)	5 (8,6)			
**EVA**			**0,001**	**<0,001**	0,147
**2 semanas**					
média ± DP	3,9 ± 3,1	2,8 ± 2,4			
mediana (p25; p75)	5 (0; 7)	2 (1; 5)			
**4 semanas**					
média ± DP	3,5 ± 2,9	1,7 ± 2,2			
mediana (p25; p75)	3 (0; 6)	1 (0; 3)			
**6 semanas**					
média ± DP	3 ± 3,2	1,4 ± 2,1			
mediana (p25; p75)	2 (0; 6)	0 (0; 2)			
**12 semanas**					
média ± DP	2,1 ± 2,9	1,3 ± 2			
mediana (p25; p75)	0 (0; 3,8)	0 (0; 2)			
**24 semanas**					
média ± DP	1,8 ± 2,8	0,9 ± 1,6			
mediana (p25; p75)	0 (0; 2)	0 (0; 2)			

**Abreviações:** AR(1), correlações autorregressivas de 1ª ordem; DP, desvio padrão; DN4, *douleur neuropathique 4 questions* ; EEG, equação de estimação generalizada; EVA, escala visual analógica; HAD, escala hospitalar de ansiedade e depressão.

**Notas:** EEG com distribuição binomial e função de ligação logito; EEG com distribuição normal e função de ligação identidade. Para ambos os modelos foi assumida matriz de correlações AR(1) entre os momentos.

A frequência de dor neuropática foi em média 17% maior no grupo placebo que o de vitamina C nas semanas avaliadas ( *p* = 0,008). A EVA foi em média 1,22 pontos maior no grupo placebo independente do momento de avaliação ( *p* = 0,001) e diminuiu em média estatisticamente ao longo das semanas independente do grupo ( *p* < 0,05), só não havendo diferença estatisticamente significativa entre avaliações consecutivas, 4 para 6 semanas, 6 para 12 semanas e 12 para 24 semanas ( *p* > 0,05), como pode ser visto na Tabela 3 .

**Tabela 3:** Resultado das comparações múltiplas entre grupos e momentos conforme diferenças encontradas para a dor neuropática e EVA

Variável	Comparação		Diferença média	Erro padrão	Valor de *p*	IC (95%)	
						Inferior	Superior
Dor neuropática (%)	Placebo e vitamina C		17,0	6,4	**0,008**	5,0	30,0
EVA	Placebo e Vitamina C		1,22	0,37	**0,001**	0,50	1,94
	2–4 semanas		0,76	0,18	**< 0,001**	0,25	1,28
	2–6 semanas		1,20	0,24	**< 0,001**	0,53	1,87
	2–12 semanas		1,71	0,27	**<0,001**	0,95	2,47
	2–24 semanas		2,08	0,29	**< 0,001**	1,26	2,90
	4–6 semanas		0,44	0,18	0,175	−0,08	0,95
	4–12 semanas		0,94	0,24	**0,001**	0,27	1,62
	4–24 semanas		1,31	0,27	**< 0,001**	0,55	2,08
	6–12 semanas		0,51	0,18	0,059	−0,01	1,02
	6–24 semanas		0,88	0,24	**0,002**	0,21	1,55
	12–24 semanas		0,37	0,18	0,443	−0,15	0,89

**Abreviaturas:** EVA, escala visual analógica; IC, intervalo de confiança.

**Notas:** Comparações múltiplas de Bonferroni.

A observação da SDCR em 12 e 24 semanas foi estatisticamente mais frequente no grupo placebo ( *p* = 0,014 e 0,007, respectivamente). O Quick-DASH em 24 semanas foi estatisticamente menor no grupo da vitamina C comparado ao placebo ( *p* = 0,040), como pode ser visto na Tabela 4 .

**Tabela 4:** Resultado das análises estatísticas de SDCR e Quick-DASH segundo grupos nos diferentes momentos de avaliação

Variável	Grupo		Total ( *N* = 122)	Valor de *p*
	Placebo ( *N* = 4)	Vitamina C ( *N* = 58)		
**SDCR 12 semanas**				
Não	57 (89,1)	58 (100)	115 (94,3)	0,014*
Sim	7 (10,9)	0 (0)	7 (5,7)	
**SDCR 24 semanas**				
Não	56 (87,5)	58 (100)	114 (93,4)	0,007*
Sim	8 (12,5)	0 (0)	8 (6,6)	
**Quick-DASH 24 semanas**				
média ± DP	15,2 ± 25,5	5,9 ± 13	10,8 ± 21	**0,040£**
mediana (p25; p75)	0 (0; 25)	0 (0; 0)	0 (0; 16)	

**Abreviaturas:** DP, desvio padrão; Quick-DASH, Quick Disabilities of the Arm, Shoulder and Hand; SDCR, síndrome da dor complexa regional.

**Notas:** *Teste exato de Fisher; # Teste da razão de verossimilhanças; £ Teste Mann-Whitney.

A gravidade da fratura, de acordo com a classificação AO, não apresentou associação estatisticamente significativa com a SDCR e dor neuropática ( *p* > 0,05).

Pacientes que apresentaram dor neuropática em 12 semanas apresentaram estatisticamente maior escore HAD ( *p* = 0,048), observando-se diferença parecida em 24 semanas, porém, não foi estatisticamente significativa ( *p* = 0,220).

## Discussão

Pacientes que receberam vitamina C apresentaram um controle superior da dor pós-operatória em comparação a aqueles que receberam placebo. Essa suplementação demonstrou reduzir significativamente a intensidade da dor, o que pode ser atribuído ao seu efeito na modulação da inflamação e na redução do estresse oxidativo. Esses resultados sugerem que a vitamina C pode ser uma intervenção eficaz para melhorar o manejo da dor no período pós-operatório, concordando com o que se encontra descrito na literatura.

Evidências indicam que a administração de ácido ascórbico (vitamina C) pode apresentar propriedades analgésicas em algumas condições clínicas. 7 Trata-se de um micronutriente essencial, com evidências que apoiam seu papel para uma infinidade de processos metabólicos relacionados à saúde mental, resposta ao estresse, formação óssea, reparo tecidual, produção de colágeno e percepção da dor. 8 14 A vitamina C influencia a resposta inflamatória, podendo modular a produção de citocinas inflamatórias e a atividade de células imunes, reduzindo inflamação associada à dor. 7

A vitamina C é um antioxidante solúvel em água com elevada concentração no sistema nervoso central, excedendo em 10 vezes a concentração sérica. 15 16 17 Trata-se de um poderoso agente antioxidante e anti-inflamatório, constituindo um cofator na esteroidogênese adrenal e biossíntese de atecolaminas. Pode também aumentar a síntese de endomorfinas e endorfina, e atua como cofator para a biossíntese de peptídeos opioides amidados. 7 8

Acredita-se que a resposta nociceptiva do ácido ascórbico é amplamente mediada pelos receptores N-metil-D-aspartato (NMDA), mais especificamente através da interação com os glutaminérgicos ionotrópicos. Existe boa evidência que revela o envolvimento dos receptores NMDA na modulação da dor, compostos que reduzem a transmissão e exercem ação antinociceptiva. 15 17 18

Conforme descrito na revisão de Fukushima e Yamazaki, o aumento da demanda do corpo por ácido ascórbico em contextos cirúrgicos é, provavelmente, devido ao estresse oxidativo. Diante disso, pacientes necessitam de doses maiores do que as recomendadas para vitamina C diária após a cirurgia, com sua administração exógena sendo associada a melhores resultados cirúrgicos. 19 Estudos mostram que sua administração também está associada a uma menor necessidade de analgésicos opioides em ambientes cirúrgicos. 20

O impacto positivo significativo da vitamina C na prevenção da dor neuropática e no surgimento da SDCR encontrado no presente estudo está de acordo com dados descritos da literatura. Os pacientes que a receberam apresentaram uma redução notável na incidência e intensidade da dor neuropática, além de uma menor ocorrência de SDCR, em comparação com o grupo placebo. Esses efeitos podem ser atribuídos à capacidade da vitamina C de reduzir a inflamação e o estresse oxidativo, fatores críticos no desenvolvimento e manutenção dessas condições dolorosas.

Radicais livres estão criticamente envolvidos na geração de dor em várias condições, incluindo a neuropática e a inflamatória. Os antioxidantes podem estar envolvidos na inibição da modulação e atenuam a alodinia mecânica induzida por lesão. Sua associação apresenta um efeito antialodínico maior no processamento da dor neuropática na medula espinhal. 21

Foi demonstrado que o ácido ascórbico protege os neurônios corticais dos efeitos tóxicos do NMDA, efeito esse mediado ao longo de seus receptores. 15 Níveis de ácido ascórbico no cérebro estão associados com a atividade desses receptores e aumentar sua concentração pode ser benéfico para pacientes em risco de complicações neurológicas. 15 17 22 23

Ensaios clínicos randomizados investigaram o efeito da vitamina C na incidência de SDCR em pacientes com cirurgia de punho e tornozelo. A dose usada nestes estudos variou de 0,2 a 1,5 g/dia por 45 a 50 dias após a cirurgia. Foi observada uma diminuição na incidência da SDRC em pacientes que receberam vitamina C, sendo as doses de ≥ 0,5 g/dia as mais eficazes. 9 24 25 26 Pesquisas anteriores indicaram que a suplementação necessária para pacientes cirúrgicos é de > 0,5 g/dia para restaurar o status normal nesses pacientes. 7 19

A suplementação de vitamina C tem sido associada com melhores resultados funcionais, inclusive diminuição da dor e do risco de SDCR após cirurgia ortopédica. 27

Contrariando os resultados apontados no presente estudo e os dados da literatura descritos anteriormente, Ekrol et al. 24 não encontraram diferença significativa no escore DASH, na incidência da SDCR, ou na consolidação das fraturas ao longo de 1 ano, concluindo que a administração de vitamina C não confere nenhum benefício a pacientes com FEDR.

Uma metanálise de sete ensaios clínicos randomizados demonstrou que a suplementação oral de vitamina C pode reduzir o risco para SDCR do tipo I, mas não melhorou os resultados funcionais em pacientes ortopédicos. 28

Uma limitação do estudo foi o número de participantes. Apesar de o cálculo amostral ter indicado a necessidade de 850 indivíduos, o presente estudo obteve resultados significativos com 122. Isso pode ser atribuído a um efeito terapêutico mais pronunciado do que o previamente estimado, à menor variabilidade intra- e intergrupos, ou à alta aderência ao protocolo de intervenção. Em determinadas situações, uma diferença clínica substancial entre os grupos pode levar à significância estatística mesmo com uma amostra menor. É importante destacar que, embora os resultados sejam promissores, essa limitação pode impactar a robustez e a generalização dos achados. Portanto, estudos com maior poder amostral são recomendados para confirmação e validação externa.

## Conclusão

O presente estudo demonstrou que a vitamina C pode ter influência positiva na prevenção da dor neuropática, da SDCR, bem como no controle da dor pós-operatória e fase inflamatória em pacientes com FEDR.
